# National Preparedness Month — September 2013

**Published:** 2013-09-06

Each September since 2004, the Federal Emergency Management Agency (FEMA) has observed National Preparedness Month. During September, FEMA and various local, state, and federal agencies encourage U.S. residents to become better prepared for emergencies and disasters. Approximately 3,000 organizations, including the American Red Cross, Citizen Corps, and CDC (1), are scheduling events and activities this month in support of the preparedness initiative.

On this 10th anniversary of National Preparedness Month, CDC also is recognizing the first decade of activity of its Emergency Operations Center (2). Staffed around the clock, 365 days of the year, the center is a state-of-the-art command facility from which scientists and emergency personnel monitor and coordinate CDC’s response to a wide range of public health threats (3).

All persons can take important steps to prepare themselves, their families, and loved ones for a possible disaster. CDC has various tools and checklists to help everyone “be ready” at home, at places of work and worship, and within the larger community (4). Additional information regarding emergency preparedness and response is available at http://www.cdc.gov/phpr.
